# Analysis of alpha-synuclein harvested from intracranial instruments used in deep brain stimulation surgery for Parkinson’s disease

**DOI:** 10.21203/rs.3.rs-4369598/v1

**Published:** 2024-05-20

**Authors:** zachary sorrentino, Joshua Riklan, Grace Lloyd, Brandon Lucke-Wold, David Mampre, Stephan Quintin, Rasheedat Zakare-Fagbamila, Megan Still, Vyshak Chandra, Kelly Foote, Benoit Giasson, Justin Hilliard

**Affiliations:** University of Florida; University of Florida; University of Florida; University of Florida; University of Florida; University of Florida; University of Florida; University of Florida; University of Florida; University of Florida; University of Florida; University of Florida

**Keywords:** Parkinson’s disease, alpha-synuclein, essential tremor, deep brain stimulation, neurodegeneration

## Abstract

Alpha-synuclein (αSyn) forms pathologic aggregates in Parkinson’s disease (PD) and is implicated in mechanisms underlying neurodegeneration. While pathologic αSyn has been extensively studied, there is currently no method to evaluate αSyn within the brains of living patients. Patients with PD are often treated with deep brain stimulation (DBS) surgery in which surgical instruments are in direct contact with neuronal tissue; herein, we describe a method by which tissue is purified from DBS surgical instruments in PD and essential tremor (ET) patients and demonstrate that αSyn is robustly detected. 24 patients undergoing DBS surgery for PD (17 patients) or ET (7 patients) were enrolled; from patient samples, 81.2 ± 44.8 μg protein (n=15) is able to be purified, with immunoblot assays specific for αSyn reactive in all tested samples. Light microscopy revealed axons and capillaries as the primary components of purified tissue (n=3). Further analysis was conducted using western blot, demonstrating that truncated αSyn (1–125 αSyn) was significantly increased in PD (n=5) compared to ET (n=3), in which αSyn misfolding is not expected (0.64 ± 0.25 vs. 0.25 ± 0.12, P = 0.046), thus showing that pathologic αSyn can be reliably purified from living PD patients with this method.

## Background

Parkinson’s disease (PD) is a neurodegenerative disorder characterized clinically by a stereotypical movement disorder and neuropathologically by the accumulation of misfolded alpha-synuclein (αSyn) within neurons and glia thought to be etiologic in neuronal dysfunction and symptom manifestation^1–4^. Symptomatically, the disease begins with a prodromal period characterized by dysautonomia and sleep disturbances thought to be consequent to brainstem manifestation of disease, subsequently the progressive movement disorder with bradykinesia and other motor symptoms linked to dopaminergic and striatal dysfunction occur, and eventually cognitive and affective decline traced to alterations in cortical function develops at end stage^3–6^. Effective treatments for PD mainly exist for alleviation of motor symptoms, with dopaminergic augmentation therapies such as levodopa and similar pharmaceuticals being first line treatment after diagnosis which occurs after classical movement disorder symptoms are evident^4^. When motor symptoms become refractory to medical management, or dyskinetic side effects from these medications are intolerable, neurosurgical treatment with deep brain stimulation (DBS) to modulate striatal circuitry involved in voluntary movement has proven to be an effective treatment for bradykinesia, tremor, rigidity, and dyskinesias^4,7^. Similarly, essential tremor (ET) is frequently treated with DBS to modulate movement circuitry through the dentato-rubro-thalamic tract (DRTT) and ventral intermediate thalamic nucleus (VIM); PD and ET differ in neuropathology in that misfolded αSyn inclusions are not abundant or causally implicated in ET^7,8^. Dopaminergic medications and DBS provide substantial relief of impaired motor function in PD, however, there currently exists no disease modifying therapies proven to prevent neuropathological PD progression, or the involvement of limbic and cortical regions associated with cognitive and behavioral decline^3,4^.

One of the major theories regarding the initiation and progression of PD symptoms and neuropathology is the prion-like spread of misfolded αSyn throughout the central nervous system^1,3^. Neuronal Lewy bodies (LBs) comprised of misfolded αSyn are pathologic hallmarks of PD, and it has been demonstrated that they arise in a roughly caudal to rostral progression throughout the brain with clinical symptoms developing that are thought to be attributable to the appearance of LBs and similar αSyn pathology in a given region^3,5,9^. Various studies have confirmed that misfolded αSyn fibrils isolated from LBs or formed in vitro when injected into animal models will induce formation of LB pathology from endogenous αSyn throughout the neuro-axis; LB development is thought to occur via spread of pathologic αSyn through interconnected neural pathways, reflecting known in vitro experimentation in which αSyn fibrils will spontaneously elongate and then fragment to form more fibrils in the presence of monomeric αSyn^1,9–12^. The appearance of LB pathology in these prion-like animal models of PD is closely linked to neuronal death, movement symptoms, and behavioral changes which suggests that preventing the prion-like spread of αSyn pathology could be key to halting the progression of PD^9,11,13,14^. While animal models are useful for undefirstanding the potential for pathologic αSyn to drive disease progression, the temporal relationship between LB formation and symptom development has not been confirmed in living humans and is only inferred from post-mortem studies. Furthermore, if αSyn misfolding is key to PD progression then there exists a need to identify which forms of misfolded αSyn should be targeted to prevent prion-like propagation to as of yet unaffected brain regions in the quest to develop disease modifying therapies for PD. Prior investigations of animal models and post-mortem human samples have shown that key biochemical differences between physiologic and misfolded αSyn are mainly in the form of post-translational modifications (PTMs), of which phosphorylation at Ser129 (pSer129) and C-terminal truncation (ΔC) are abundant; these modifications are thought to modulate the propensity of αSyn to misfold and form inclusions which may prove useful in evaluating therapeutic targets^1,15–20^.

Herein, we seek to confirm that pathologic αSyn is developing in the frontal cortex of living PD patients prior to the appearance of significant cognitive symptoms, and characterize which forms of pathologic αSyn are present at this early stage that may be amenable to therapeutic intervention. We aim to accomplish these objectives first through development of a protocol to isolate αSyn from surgical instruments that traversed the frontal cortex in PD patients undergoing DBS, and ET patients treated with DBS as a control group. Secondly, we perform a pilot analysis on the forms of αSyn purified and detect differences between the PD and ET patients.

## Methods

### Regulatory approval and participants

All study activities were conducted in accordance with University of Florida (UF) Gainesville Health Science Center Institutional Review Board (IRB-01) issued approval (IRB202300741). Study procedures, risks, benefits, and alternatives were explained to all participants, and informed consent was obtained according to IRB protocol after being provided written documentation of study participation. A waiver for written consent was allowed given that the only record linking the subject and the research would be the consent document and the principal risk would be potential harm resulting from a breach of con dentiality. All protocols were followed in accordance with UF IRB policies and regulations to conduct safe research with human participants.

This study on the feasibility of collection and analysis of residual brain tissue on deep brain stimulation (DBS) surgical instruments, particularly cannulas and microelectrodes, was conducted with de-identified samples without any HIPPA identifiers. Surgical instruments from DBS procedures performed by two functional neurosurgeons for patients with Parkinson’s disease (PD) or essential tremor (ET) conducted at UF over a 6-month period were collected. Inclusion criteria were: patients between the ages of 18 and 89 years old undergoing a DBS procedure performed for a pre-operative diagnosis of PD or ET. Diagnosis was made or confirmed by movement disorder neurologists at the University of Florida Norman Fixel Institute for Neurological Diseases. Selected patients were recommended for treatment with DBS by a multidisciplinary board of clinicians as previously described for both PD and ET^21,22^. Included subjects underwent staged bilateral DBS lead implantation into either the ventral intermediate thalamic nucleus (VIM) for patients with ET, or the subthalamic nucleus (STN), globus pallidus internus (GPI), or VIM for patients with PD. Surgical instruments from a procedure were excluded if the designated investigator performing data analysis was a surgeon in the DBS procedure, if surgical instruments were contaminated during the procedure (for example, inadvertently dropped on the floor), or if surgical instruments were visibly bloody. The only demographic data collected from patients were their pre-operative diagnosis and surgical target for the DBS procedure they underwent (Table 1).

### Surgical details and collection of surgical instruments

DBS target selection and surgical protocol was performed as previously described^23^. After discussion of risks, benefits, and alternatives of the procedure, surgical consent was obtained. Patients underwent placement and registration of a stereotactic head ring for DBS lead targeting. All planned surgical trajectories traversed the posterior region of the frontal lobe anterior to the primary motor cortex, typically through the superior or middle frontal gyrus and subsequently through the corona radiata and deep white matter until the VIM, GPI, or STN was encountered as planned. A high-resolution contrasted MRI sequence was used to plan trajectories with care to avoid the ventricles, cortical vessels, and deep parenchymal vessels. After trajectory planning, patients were brought to the operative suite where the scalp was prepared and draped in the usual sterile fashion. Local anesthesia was utilized, and patients remained awake for the duration of the procedure. The stereotactic trajectory was marked, scalp and periosteal tissue divided in a linear incision, and a burr hole made with a high-speed drill. Hemostasis was obtained with bipolar electrocautery, and the dura mater overlying the target brain was cauterized and incised. A small entry point through the leptomeninges was created, and the standard 1.4mm diameter guide cannula with stylet (Alpha Omega, Nazareth, Israel) introduced into the parenchyma to a variable depth of ~ 3 to 6 cm depending on target. The inner stylet of the cannula was removed, and a shielded “NeuroProbe” electrode (Alpha Omega, Nazareth, Israel) introduced through the cannula and further into the parenchyma an additional 2.5 cm during microelectrode recordings. Intra-operative microelectrode recordings were performed by the neurology team to define the target region, optimizing lead placement. Once concluded, the microelectrode was removed and the cannula was further advanced 2.5 cm to the target structure (VIM, GPI or STN). The permanent DBS lead was implanted with further macrostimulation testing, and then the lead was secured in place and cannula removed. The cannula and microelectrode were placed into their original sterile packaging by members of the surgical team using sterile technique and placed in storage at 4°C until residual tissue purification from these devices was performed within 6 hours of collection as described below.

### Purification of residual tissue from cannulas and electrodes

**Figure 1:**
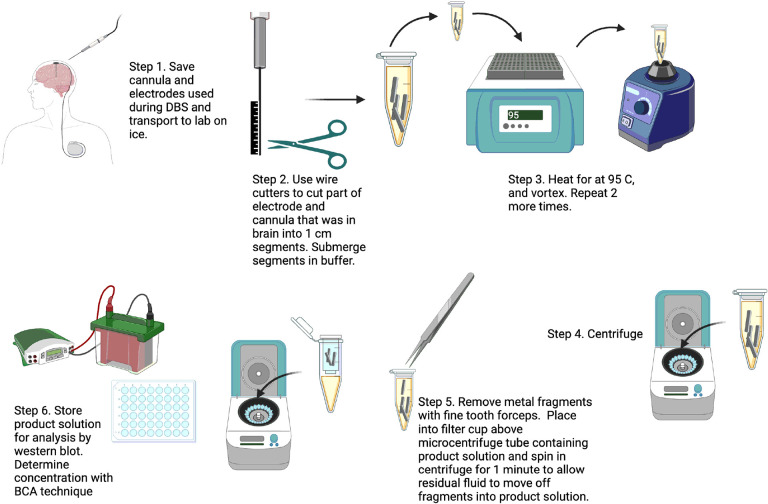
Graphical description of the purification process using SDS by which PD and ET surgical instruments were processed and purification of protein with subsequent western blot analysis conducted. Modified versions of this procedure were used for PBS based purification as described. Figure created using Biorender.com.

Cannulas and microelectrodes were transferred on ice in sterile packaging from the operative suite to laboratory facilities for purification of tissue. For each subject, the cannula (including stylet) and microelectrode were first inspected and the approximate portion of each which had been placed intraparenchymal were marked and cut from the remaining portion with wire cutters. The intraparenchymal portions of the cannula and microelectrode were then further sectioned into ~ 1 cm portions and placed into a 1.5 mL microcentrifuge tube containing 150 μL of extraction buffer (0.2% sodium dodecyl sulfate (SDS), 1% Triton X-100 in 1x phosphate buffered saline (PBS), pH 7.4) at 25°C. Samples were then vigorously shaken on a vortex mixer for 5 minutes and then heated at 95°C for 5 minutes to solubilize and denature residual tissue on the cannula and microelectrode sections; this procedure was repeated three times to ensure maximal extraction of tissue. Samples were then centrifuged at 21,000 × g for 10 minutes at 25°C. Cannula and microelectrode fragments were removed from the microcentrifuge tube and placed into a miniprep column (filter removed; Promega) that was then attached to the top of the microcentrifuge tube; the microcentrifuge tube with a column in place was further centrifuged at 1,500 × g for 2 minutes at 25°C to drive residual liquid sample from fragments into the sample solution before discarding (Fig. 1). The sample was then stored at −80°C until subsequent analysis. Concentrations of samples were determined using the bicinchoninic acid (BCA) assay (Pierce, Waltham, MA) with bovine serum albumin (BSA) as the standard.

### Retrieval and preparation of reference autopsy tissue

Human brain tissue was obtained through the University of Florida Neuromedicine Human Brain Tissue Bank in accordance with institutional review board approval. Post-mortem pathological staging and diagnoses were made according to respective neuropathological criteria for Lewy body dementia (LBD)^24^. Un fixed samples of cingulate cortex from two post-mortem cases of LBD were retrieved and homogenized in 500 μL of extraction buffer detailed above. Case 1 was age 68 at death and case 2 was age 83 years at death, both with Braak stage VI of LBD pathology. Samples were then similarly shaken on a vortex mixer for 5 minutes and then heated at 95°C for 5 minutes to solubilize and denature tissue; this was repeated 3 times.

### SDS polyacrylamide gel electrophoresis and Coomassie blue staining

Sample buffer (10 mM Tris, pH 6.8, 1 mM ethylenediaminetetraacetic acid (EDTA), 40 mM dithiothreitol (DTT), 0.005% Bromophenol Blue, 0.0025% Pyronin Yellow, 1% SDS, 10% sucrose) was added to all samples which were subsequently boiled for 10 minutes. 20 μg of sample from cases 1 and 2 of autopsy tissue and 20 μg of two representative PD DBS samples were resolved on SDS polyacrylamide gel electrophoresis (SDS-PAGE) using 15% polyacrylamide gels. Gels were stained with Coomassie blue R-250 to visualize protein and destained in 25% isopropanol/10% acetic acid as previously described^12^.

### Antibodies utilized for western blotting and immunofluorescence

For western blotting, anti-phosphorylated Ser129 (pSer129) αSyn rabbit monoclonal antibody EP1536Y was obtained from Abcam (Cambridge, MA). SNL-4 is a rabbit polyclonal antibody specific for αSyn residues 2–12^25^. 3H11 is a mouse monoclonal antibody specific for the 43–63 residues of αSyn^2,26^. 5C1 is a mouse monoclonal antibody specific for αsyn C-terminally truncated at residue 125^16^. For immunofluorescence, neuronal specific rabbit anti-βIII tubulin antibody (T2200) was obtained from Sigma-Aldrich (St. Louis, MO).

### Western blotting

Representative samples were chosen, and 5 μg of each sample (5 PD samples and 3 ET samples per blot) were loaded onto 15% polyacrylamide gels and resolved by SDS-PAGE, followed by electrophoretic transfer onto 0.2 μm pore nitrocellulose membranes (Bio-Rad, Hercules, CA) in carbonate transfer buffer (10 mM NaHCO3, 3 mM Na2CO3, pH 9.9) as previously described^12^. Nonspecific binding of antibodies was blocked with 5% dry milk/Tris buffered saline (TBS) and membranes were incubated overnight at 4°C with primary antibody diluted in the same block solution. Membranes were washed in TBS and then incubated in goat anti-rabbit or goat anti-mouse secondary antibodies conjugated to horseradish peroxidase (Jackson Immuno Research Labs, Westgrove, PA) and diluted in block solution for 1 hour at 25°C; immunocomplexes were detected with Western Lightning-Plus ECL reagents (PerkinElmer, Waltham, MA) followed by chemiluminescence detection (PXi, Syngene, Frederick, MD). Densitometry was performed via ImageJ software (NIH, Bethesda, MD) to quantify the signal for each sample. Western blot images are displayed with increased brightness and contrast for ease of viewing, with originals in supplementary information.

### Purification of residual tissue for Hematoxylin & Eosin staining

For 5 DBS procedures on PD patients, tissue was purified from cannulas and microelectrodes in modified, non-denaturing conditions using PBS which allowed for hematoxylin & eosin (H&E) staining in 3 of the cases. Cannulas and microelectrodes were sectioned into ~ 1 cm fragments and placed into microcentrifuge tubes containing 150 μL of 1x PBS on ice. Samples were homogenized via manual pipetting, and then centrifuged at 1,500 × g for 5 minutes at 25°C. Cannula and microelectrode fragments were removed from the microcentrifuge tube and placed into a miniprep column and then centrifuged at 1,500 × g for 2 minutes at 25°C similar to the protocol detailed previously. For three of the samples, liquid sample was then transferred to a glass slide and 50 μL of 10% paraformaldehyde in 1x PBS was added for fixation overnight at 4°C. Samples were then rinsed in 1x PBS and placed into hematoxylin for staining, followed by aqueous washes. Samples were then stained in eosin followed by dehydration in ethanol, then xylene, then mounting and cover slipping. All slides were digitally scanned using an Aperio ScanScope CS instrument (40× magni cation; Aperio Technologies Inc., Vista, CA), and images of representative areas of staining were captured using the ImageScope software (40× magnification; Aperio Technologies Inc.).

For the 2 PD samples not used for H&E, PBS purified tissue was homogenized and denatured through sequential boiling (95°C for 5 minutes) and shaking on a vortex mixer for 5 minutes repeated 3 times. This procedure was also utilized for 50 μL of tissue from case PD 20 for which the remainder of sample was used for H&E. Protein amount was then determined as described above using BCA assay, which was multiplied threefold for case PD 20 to account for the 100 μL used for H&E.

### Immunofluorescence

For two DBS procedures on PD patients, tissue was purified from cannulas and microelectrodes and transferred to glass slides with paraformaldehyde fixation with the same protocol used for the H&E staining. Samples were then rinsed in 1x PBS and incubated with primary antibody overnight (4°C) diluted in 5% FBS/0.1M Tris (pH 7.6) followed with subsequent incubation for 1 hour at 25°C using secondary antibody (diluted in 5% FBS/0.1M Tris, pH 7.6) conjugated to Alexa-594 (Invitrogen). Samples were stained with 5 μg/mL 4’,6-diamindino-2-phenylindole (DAPI). Lastly, the sections were cover-slipped with Fluoromount-G (SouthernBiotech) and visualized using an Olympus BX51 microscope mounted with a DP71 Olympus digital camera to capture images at 40x magnification.

### Quantitative analysis

All statistical tests and resulting graphs were conducted and created using GraphPad Prism (GraphPad software, La Jolla, CA). Comparison of protein amount between PD and ET samples purified using an SDS method was conducted using an unpaired T-test. Similarly, comparison of protein amount between all samples purified with SDS compared to the 3 using PBS was conducted using an unpaired T-test. For western blot data, bands were quantified using ImageJ software (NIH, Bethesda, MD) and densitometric comparisons between PD and ET bands for each antibody conducted using an unpaired T-test. For all gures, conventional terminology used for P-values is NS = no significance, * = P ≤ 0.05, ** = P ≤ 0.01, *** = P ≤ 0.001, **** = P ≤ 0.0001.

## Results

### Cohort Characteristics and tool collection

From May to November 2023, patients with a pre-operative diagnosis of PD or ET undergoing DBS surgery due to movement symptoms refractory to maximum medical treatment were invited to participate in this study. 24 patients met inclusion criteria and consented to enrollment; the cohort comprised of 17 PD and 7 ET patients with each undergoing staged bilateral DBS lead implantation into either the VIM, GPI, or STN through a trans-frontal cortex trajectory (Table 1). No patients declined to participate. Per IRB criteria, no other demographic information was collected from participants outside of pre-operative diagnosis and surgical target. Upon collection of cannulas and electrodes, two of the samples were visibly contaminated with blood (PD 1 and PD 17) and excluded from further analysis; these samples were instead utilized for method development and proof of concept for experimental procedures (Table 1).

### Protein purification from surgical instruments and profile comparison with post-mortem tissue

**Figure 2:**
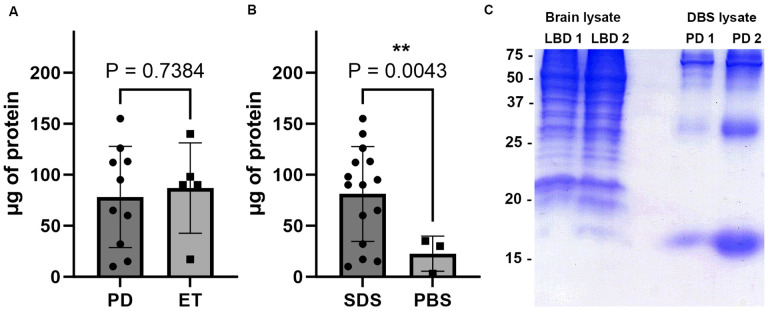
Analysis of protein obtained from DBS surgical instruments and comparison with whole brain lysate A. Amount of protein purified in μg using SDS based method from PD (n = 10) vs ET (n = 5) samples, error bars = std. B. Amount of protein purified in μg from samples using SDS based method (n = 15) vs PBS based method (n = 3), error bars = std. C. Coomassie stained SDS-PAGE with 20 μg sample of whole brain lysate from two cases of LBD cingulate cortex on the left and 20 μg of DBS purified sample from two PD cases on the right. Protein size in kDa is shown, with expected prominent band consistent with αSyn at ~17 kDa.

Residual tissue on surgical instruments from 10 PD and 5 ET surgeries were purified using the SDS based method described (Fig. 1). An additional 3 cases of PD had sample purified using the PBS based method described. Protein amount was determined using the BCA assay for each sample. On comparison between protein amount purified from PD and ET samples using the SDS based method, the 10 PD cases had 78.3 ± 47.0 μg protein per sample while the 5 ET cases had 87.0 ± 39.6 μg protein per sample which on t-test comparison was not significantly different (P = 0.738, Fig. 2A). Overall, 81.2 ± 44.8 μg protein per sample was able to be purified from the 15 surgical instruments using a SDS method, whereas from the 3 surgical instruments from which protein was collected using a PBS based method there was 22.6 ± 14.0 μg protein per sample which was significantly different on t-test (P = 0.0043, Fig. 2B).

To further characterize protein extracted from DBS samples, 15% acrylamide SDS-PAGE with Coomassie blue staining was conducted using 2 PD DBS samples and 2 post-mortem LBD cingulate cortex samples (mix of grey and white matter) for comparison (Table 1, Fig. 2C). 20 μg of sample were loaded into each well. On comparison, the 2 LBD cortex samples contained a dense array of protein bands from 75 to 37 kDa, and many additional bands from 37 to 15 kDa (Fig. 2C). In contrast, the 2 PD DBS samples display fewer bands from 75 to 37 kDa, with a major band at ~ 30 kDa and one prominent ~ 17 kDa band consistent with the mobility of αSyn (Fig. 2C)^1^. Overall, while multiple protein bands are present on SDS-PAGE of these DBS samples, the number of bands and diversity of band size is much less than that from the total brain samples.

### Identification of sample cellular characteristics upon H&E and immunofluorescent staining

**Figure 3:**
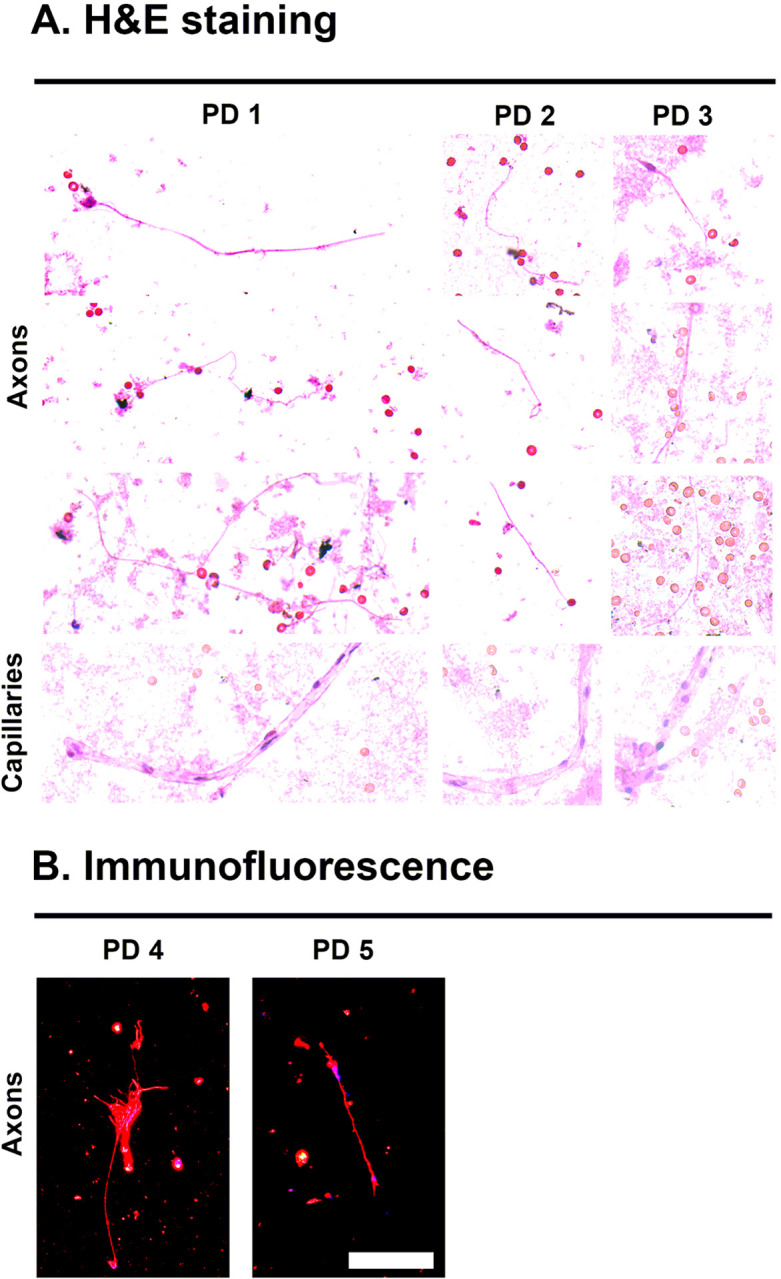
Microscopic characteristics of purified DBS samples. A. H&E light microscopy representative findings from three cases of PD DBS sample. Demonstrated are ~1 um filamentous structures consistent with axons and larger cylindrical structures the width of a red blood cell consistent with capillaries. Scale reference are red blood cells with width ~ 6 μm. B. Immunofluorescent microscopy representative findings from two cases of PD DBS sample. Red = βIII-tubulin staining on presumed axons and blue = DAPI. Scale bar = 10 μm.

In order to further characterize any cellular components in the DBS samples, tissue was purified from 3 of the PD DBS samples (Table 1) under non-denaturing conditions use the PBS method described. Liquid sample was transferred to glass slides, fixed in formalin and subject to H&E staining and then qualitative analysis with light microscopy (Fig. 3A). In all 3 samples, linear eosinophilic filamentous structures of ~ 1 μm in length were readily observed which are consistent with axons (Fig. 3A). Additionally, tubular structures of a width slightly larger than a red blood cell with interspersed hematoxylin staining are present in all 3 samples which are consistent with capillaries (Fig. 3A). Red blood cells are also found intermittently throughout the samples which are used as a size standard given known width of 6 μm. No fully intact neurons or glial cells were seen in the samples, suggesting that most tissue on the DBS samples are from axons, capillaries and blood cells (Fig. 3A). In order to further confirm that the abundant filamentous structures seen are axons, immunofluorescent staining with neuron specific βIII-tubulin was performed on 2 of the PD DBS samples which demonstrated avid fluorescence on the axons (Table 1, Fig. 3B).

### Analysis of total, phosphorylated and truncated αSyn from samples by western blotting

**Figure 4:**
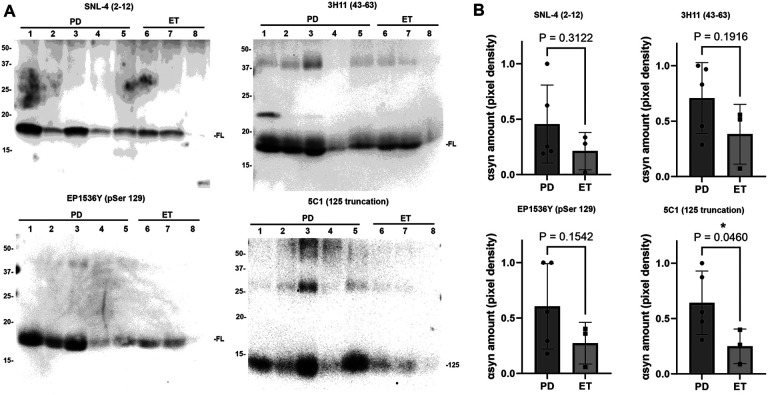
Western blot immunochemical detection of αSyn. A. DBS samples purified using SDS were obtained from ve PD samples (lanes 1–5) and three ET samples (lanes 6–8) and subject to immunoblotting comparison of αSyn species using a panel of four antibodies which are indicated. 5 μg of sample are loaded in all lanes for all samples. Antibody SNL-4 specific for residues 2–12 of αSyn demonstrates αSyn detection at ~17 kDa for all samples, antibody 3H11 specific for residues 43–63 of αSyn demonstrates αSyn along with additional heavier bands that may be oligomers. Antibody EP1536Y specific for pSer129 αSyn detects this epitope at ~17 kDa in all samples, and antibody 5C1 specific for truncated αSyn at 125 displays detection of ~14 kDa band preferentially in the PD samples. Band size in kDa is shown. B. Densitometric analysis of band detection in PD (n = 5) and ET (n = 3) samples with each of the four antibodies utilized; comparison using T-test and associated P-value displayed. 5C1 has significantly enhanced detection of truncated αSyn at 125 in PD samples compared with ET. Western blot images are displayed with increased brightness and contrast for ease of viewing, with originals in supplementary information.

To determine whether pathologic αSyn can be detected from PD DBS samples, and perform preliminary comparisons with ET in which αSyn is expected to be present only in physiologic form, western blot analysis with various αSyn antibodies was conducted using 5 PD samples and 3 ET samples (Table 1). Samples were purified from DBS surgical instruments in each case using the SDS method described above, and 5 μg of each sample was loaded on 15% polyacrylamide gels and analyzed by immunoblotting with various antibodies specific for total αSyn (SNL-4 and 3H11), pSer129 αSyn (EP1536Y), and x-125 carboxyl-truncated αSyn (5C1) (Fig. 4A). Upon detection with each αSyn antibody, signal intensity for each band was quantified using ImageJ and unpaired T-tests used to compare signal between the PD and ET samples for each antibody (Fig. 4B).

To compare total αSyn between PD and ET, antibody SNL-4 was applied which is specific for αSyn residues 2–12^25^. Avid detection was visualized amongst all PD and ET samples, with the expected ~ 17 kDa αSyn band labeled and no significant difference in normalized signal intensity between PD and ET (0.45 ± 0.31 for PD vs. 0.21 ± 0.14 for ET, P = 0.39). A second total αSyn antibody, 3H11 which is specific for the 43–63 residues of αsyn, was utilized as it has been demonstrated to detect possible additional αSyn species^2,26^. On comparison between PD and ET labeling with 3H11, 1–2 additional larger kDa bands are seen across samples, however on normalized signal intensity of the ~ 17 kDa αSyn band there was no significant difference (0.70 ± 0.28 for PD vs. 0.40 ± 0.22 for ET, P = 0.19). Anti-phosphorylated Ser129 (pSer129) antibody EP1536Y was used to detect αSyn with the pSer129 post-translational modification, which is a common immunohistochemical marker of pathologic αSyn and is known to label LBs^1^. On comparison between PD and ET labeling with EP1536Y, normalized signal intensity of the ~ 17 kDa αSyn band between samples trended towards significance but was not significantly different between PD and ET (0.60 ± 0.34 for PD vs. 0.26 ± 0.15 for ET, P = 0.15).

5C1 is an antibody specific for αSyn C-terminally truncated at residue 125; this antibody has previously been shown to detect αSyn containing residues 1–125 but not the full-length form^16^. C-terminally truncated αSyn is known to pathologically aggregate more readily than physiologic αSyn, and it is readily detected in pathology laden brain regions in LBD^1,16^. On comparison between PD and ET labeling with 5C1, a ~ 14 kDa band was seen which is consistent with the 1–125 truncated form of αSyn, and normalized signal intensity of this band between samples demonstrated a significant difference between PD and ET (0.64 ± 0.25 for PD vs. 0.25 ± 0.12 for ET, P = 0.046).

## Discussion

This study demonstrates a method by which αSyn can be safely purified from disposable surgical instruments used in DBS surgery which allows for evaluation of pathologically misfolded forms of αSyn from the brains of living patients. By utilizing samples from patients who underwent DBS for PD or ET, this method allows for comparison of αSyn from PD, in which pathologic modifications are expected, with αSyn from ET where LBs are rare and αSyn is assumed to be in its physiologic form^8^. Herein, we first demonstrated that αSyn can be readily purified from cannulas and microelectrodes utilized in DBS that traversed the frontal cortex and deep white matter in PD and ET, with the major component of residual parenchyma on these surgical instruments likely being axons and capillaries. In comparison with brain lysate from post-mortem samples, this residual parenchyma from living patients does not have as many protein bands on SDS-PAGE which in conjunction with microscopy findings suggests that intact neuronal soma are not present on these surgical instruments but axonal and synaptic proteins such as αSyn are abundant and can be optimally and safely studied from the brains of living patients with this method. Using the SDS based method in this study, the amount of protein purified on the order of ~ 80 μg per sample is sufficient for multiple immunochemical assays, and by using less harsh purification methods there is a four-fold reduction in protein purified but preservation of cytoarchitecture and higher order protein structure. Utilizing the residual tissue on DBS surgical instruments has allowed us for the first time to systematically and safely analyze αSyn from brain tissue of living patients. Long term follow-up of patients will conceivably allow for larger studies in which progression of cognitive dysfunction can be correlated with the presence of various forms of pathologic αSyn.

Candidates for DBS surgery are screened by an interdisciplinary team for cognitive and psychiatric decline prior to treatment, and surgery is not offered if these symptoms are advanced due to concern for precipitating post-operative declines^21,22^. Given that PD patients undergoing DBS typically have advanced motor symptoms but still mild cognitive symptoms, analysis of αSyn from their neocortical regions traversed by surgical surgical instruments used in DBS may reveal the presence of pathologic αSyn prior to neuronal dysfunction which could represent a valuable therapeutic target to prevent progression of disease. Herein, analysis of total αSyn between PD and ET samples was not significantly different between the diseases which is expected as αSyn is an abundant neuronal protein; even in diseased parenchyma, only a small portion of αSyn is in a pathologically insoluble state^27^. αSyn phosphorylated at Ser-129 trended towards being significantly increased in PD samples; although pSer129 is one of the most common markers of LB pathology, it has previously been demonstrated that physiologic αSyn can harbor this modification which limits its utilization for diagnostics and potential therapeutic purposes^27,28^. Additionally, it is unclear whether pSer129 develops late in LB pathology as a protective mechanism versus early in a pathologic, causative role as studies have been mixed regarding its propensity to induce αSyn aggregation^15,28^. These findings of equivocal differences in pSer129 αSyn between PD and controls in living patients are in line with literature on detection of pSer129 from CSF^28–30^

C-terminally truncated αSyn with terminal residue at 125 (ΔC-125 αSyn) was significantly increased in PD compared to ET samples in this study. ΔC-125 αSyn was readily detected in the PD samples using the 5C1 specific antibody which has previously been shown to bind only the truncated form of αSyn and preferentially in diseased tissue, compared to samples without pathologic αSyn^16^. The increased presence of this ΔC-125 αSyn in these PD samples may have important therapeutic implications, as it has previously been shown to more readily assemble into pathologic amyloidogenic fibrils compared to full-length αSyn which highlights its potential as a catalyst for prion-like spread of disease^1,12^. Its presence in neocortical regions prior to cognitive decline could conceivably be leveraged as a target to slow disease progression if depleted, or if its initial formation is prevented.

Limitations of this study include only analyzing one of the multiple forms of truncated αSyn known to exist in synucleinopathies due to sample limitations in this exploratory study^1,16^, a lack of demographic data due to privacy limitations, and a limited sample size. The methods developed and findings in this study will be useful for longitudinal studies in which modified species of αSyn and other axonal proteins such as tau can be collected from patients where clinical development of cognitive symptoms can be tracked and correlated with the forms of pathologic proteins present in neocortical regions. This could then inform therapeutic target selection and enhance undefirstanding of the pathophysiologic processes underlying the progression of neuropathologic disease.

## Conclusions

This study demonstrates a reliable and safe method by which pathologic αSyn can be safely purified from brain tissue for the first time in living patients with PD. In particular, truncated αSyn is detected from neocortical regions in patients who do not yet have significant cognitive symptoms which suggests that pathologic αSyn accumulation precedes symptom development which has not previously been confirmed in living patients. Given the widespread usage of DBS to treat PD and patient long-term follow-up after treatment, detected forms of αSyn from these patients could conceivably be correlated with symptomatic progression in patients to identify therapeutic targets and prognostic markers. Furthermore, confirmation of neuropathologic markers from PD patients could be important in clinical trial enrollment and differentiation of PD from other movement disorders.

## Figures and Tables

**Table 1.** Summary of samples from PD or ET patients included in this study. Clinical diagnosis, DBS target, purification method, study use, and μg of protein purified from each sample (if pertinent) are listed. Each sample was used for only certain experimental assays which are listed; μg indicates the sample was used for detection of total protein purified, western indicates inclusion in western blot assays, “blue” means samples were used for Coomassie blue SDS-PAGE, H&E and IF (immunofluorescence) samples were used for microscopy studies, and “proof of concept” samples were used for method development. PD, Parkinson’s disease; ET, essential tremor; STN, subthalamic nucleus; VIM, ventral intermediate nucleus; GPi, Globus pallidus internus; SDS, sodium dodecyl sulfate; PBS, phosphate buffered saline; H&E, hematoxylin and eosin; IF, immunofluorescence.

Sample	Diagnosis	DBS target	Purification type	Study use	μg protein
1	PD	STN	SDS	Proof of concept	-
2	ET	VIM	SDS	μg	17
3	PD	STN	SDS	μg, western	113
4	PD	STN	SDS	μg	32
5	PD	STN	SDS	μg, blue	60
6	PD	STN	SDS	μg, blue, western	112
7	ET	VIM	SDS	μg, western	90
8	PD	GPI	SDS	μg, western	155
9	ET	VIM	SDS	μg, western	90
10	PD	STN	SDS	μg	95
11	PD	GPI	SDS	μg, western	126
12	ET	VIM	SDS	μg, western	140
13	PD	STN	SDS	μg, western	65
14	PD	VIM	SDS	μg	15
15	PD	GPI	SDS	μg	10
16	ET	GPI	SDS	μg	98
17	PD	STN	PBS	Proof of concept	-
18	PD	STN	PBS	μg	35
19	PD	GPI	PBS	μg	3
20	PD	GPI	PBS	H&E, μg	30
21	ET	VIM	PBS	H&E	-
22	ET	VIM	PBS	H&E	-
23	PD	STN	PBS	IF	-
24	PD	STN	PBS	IF	-

## Data Availability

data is available upon request from the corresponding author (Zachary Sorrentino) to aid in interpretation and verification of research contained herein.
